# Supplementary material for: Patterns of Intron Gain and Loss in Fungi
Source: PLoS Biol. 2004 Nov 30;2(12):e422. doi: 10.1371/journal.pbio.0020422 (PMC532390; doi:10.1371/journal.pbio.0020422)
Supplement: Table S1 — Also available at http://genes.mit.edu/NielsenEtAl/. (4.3 MB ZIP). [file pbio.0020422.st001.zip › NielsenEtAl/html/1241.html]

AN8258.1.NCU03623.1.MG04081.1.FG05977.1


```
 CLUSTAL W (1.82) Multiple Sequence Alignments - Introns Inserted


Sequence 1: NCU03623.1	166 aa
Sequence 2: FG05977.1	166 aa
Sequence 3: MG04081.1	166 aa
Sequence 4: AN8258.1	166 aa
Alignment Length: 167 aa
Number Identitical Residues: 119 aa
Alignment Score (without introns) 5541


MG04081.1 	MAQSTAHRRLLQEYRALTNNPPDGITAGPVNEDDLLHWEAMIQGPEGTPFEGGVFPAELR
NCU03623.1	MSLNVAQRRLLQEYRALTNNPPEGITAGPISEDDLLHWECLIQGPEGTPFEGGVFPAELK
FG05977.1 	MAQSTAHRRLLQEYRALTNNPPEGITAGPISEDDLLHWECLIQGPEGTPFEGGVFPAELK
AN8258.1  	MS-SMTQNRLFREYKTLSTNPPDGITAGPVSEDDMFHWEALIQGPEGTPYEGGVFAAELK
          	*: . ::.**::**::*:.***:******:.***::***.:********:*****.***:

MG04081.1 	FPKDYPLAPPTMKFLG-EIWHPNV1YPSGLVCISILHPPGDDPNHYEHASERWSPIQSVE
NCU03623.1	FPNDYPHMPPTMKFLG-DIFHPNV1YPSGLVCISILHPPGDDPNHYETASERWSPIQSVE
FG05977.1 	FPKDYPLAPPSMRFLA-DVWHPNV1YPSGLVCISILHPPGDDPNHYEHASERWSPIQSVE
AN8258.1  	FPKDYPLSPPTMKFVGGGVWHPNV1YPNGTVCISILHPPGDDPNHYEHASERWSPIQSVE
          	**:***  **:*:*:.. ::**** **.* ***************** ************

MG04081.1 	KILISVMSMLAEPNDESPANVEAAKMWRERRADYEQKVRDSVRRSLGL
NCU03623.1	KILLSVMSMLAEPNDESPANVEAAKMWREKRSEYEARVKASVRASLGL
FG05977.1 	KILISVMSMLAEPNDESPANVEAAKMWRERRTEYENKVREGVRRMLGL
AN8258.1  	KILISVMSMLAEPNDESPANVEAAKMWRERRAEYERKVREEVRKGLGL
          	***:*************************:*::** :*:  **  ***
```
